# Population PK/PD modelling of meropenem in preterm newborns based on therapeutic drug monitoring data

**DOI:** 10.3389/fphar.2023.1079680

**Published:** 2023-03-15

**Authors:** Sergey Zyryanov, Irina Bondareva, Olga Butranova, Alexandra Kazanova

**Affiliations:** ^1^ Department of General and Clinical Pharmacology, Peoples’ Friendship University of Russia (RUDN University), Moscow, Russia; ^2^ State Budgetary Institution of Healthcare of the City of Moscow “City Clinical Hospital No. 24 of the Moscow City Health Department, Moscow, Russia

**Keywords:** meropenem, therapeutic drug monitoring, preterm newborns, pharmacokinetics -, pharmacodynamics

## Abstract

**Background:** Preterm neonates rarely participate in clinical trials, this leads to lack of adequate information on pharmacokinetics for most drugs in this population. Meropenem is used in neonates to treat severe infections, and absence of evidence-based rationale for optimal dosing could result in mismanagement.

**Aim:** The objective of the study was to determine the population pharmacokinetic (PK) parameters of meropenem in preterm infants from therapeutic drug monitoring (TDM) data in real clinical settings and to evaluate pharmacodynamics (PD) indices as well as covariates affecting pharmacokinetics.

**Materials and methods:** Demographic, clinical and TDM data of 66 preterm newborns were included in PK/PD analysis. The NPAG program from the Pmetrics was used for modelling based on peak-trough TDM strategy and one-compartment PK model. Totally, 132 samples were assayed by high-performance liquid chromatography. Meropenem empirical dosage regimens (40–120 mg/kg/day) were administered by 1–3-h IV infusion 2–3 times a day. Regression analysis was used to evaluate covariates (gestation age (GA), postnatal age (PNA), postconceptual age (PCA), body weight (BW), creatinine clearance, etc.) influenced on PK parameters.

**Results:** The mean ± SD (median) values for constant rate of elimination (Kel) and volume of distribution (V) of meropenem were estimated as 0.31 ± 0.13 (0.3) 1/h and 1.2 ± 0.4 (1.2) L with interindividual variability (CV) of 42 and 33%, respectively. The median values for total clearance (CL) and elimination half-life (T1/2) were calculated as 0.22 L/h/kg and 2.33 h with CV = 38.0 and 30.9%. Results of the predictive performance demonstrated that the population model by itself gives poor prediction, while the individualized Bayesian posterior models give much improved quality of prediction. The univariate regression analysis revealed that creatinine clearance, BW and PCA influenced significantly T1/2, meropenem V was mostly correlated with BW and PCA. But not all observed PK variability can be explained by these regression models.

**Conclusion:** A model-based approach in conjunction with TDM data could help to personalize meropenem dosage regimen. The estimated population PK model can be used as Bayesian prior information to estimate individual PK parameter values in the preterm newborns and to obtain predictions of desired PK/PD target once the patient’s TDM concentration(s) becomes available.

## Introduction

Neonatal infections and sepsis are still among the leading factors of neonatal morbidity and mortality in the world (Perin et al., 2022). Age-standardized incidence rate of neonatal infections revealed increase for the period 1990–2019 years with estimated annual percentage change 0.46 (95% credit interval, CI: 0.43–0.48) (Ou et al., 2022). Safe and effective management of severe infections and sepsis in neonates is a challenge for clinical practice. Meropenem is a broad-spectrum beta-lactam antibiotic which is effective in treatment of severe infections of various locations, including pneumonia, meningitis, complicated skin and soft tissues infections, intra-abdominal infections. Meropenem can be used in children including neonates in order to prevent sepsis, since it has such benefits as satisfactorily safety profile in this age group and possibility to be used as monotherapy (Cohen-Wolkowiez et al., 2012; Cao et al., 2022). In neonates, meropenem is typically reserved for nosocomial infections caused by multidrug-resistant Gram-negative bacteria being a life-saving agent in infections caused by *Pseudomonas aeruginosa, Acinetobacter baumannii* (Lutsar et al., 2020).

Dosing regimens of meropenem in neonates, including patients < 3 months, vary in different recommendations, including US Food and Drug Administration (FDA) label (MERREM IV, 2022) and UK Electronic Medicines Compendium (EMC) (Electronic medicines compendium, 2022). Neonates are highly heterogeneous population in respect to organs and systems maturation, which affects drugs pharmacokinetics and thus, drugs exposure. That’s why population pharmacokinetic studies of meropenem in neonates and infants and studies based on TDM are still used for optimization of dosing regimens in such a vulnerable category of patients (Ohata et al., 2011; Maimongkol et al., 2022; Raza et al., 2022). There are few meropenem PK studies including preterm newborns (van den Anker et al., 2009; van Enk et al., 2001; Germovsek et al., 2018; Smith et al., 2011; Lima-Rogel et al., 2021; Bradley et al., 2008; Padari et al., 2012), and further investigation of pharmacokinetics in this population is still required.

Bactericidal activity of meropenem is time-dependent and correlates with the percentage of time that free-drug concentrations are higher than the minimum inhibitory concentration, MIC (%T > MIC). To treat most infections the desired PK-PD index of meropenem is the unbound plasma meropenem concentration above the minimum inhibitory concentration (MIC) (fT > MIC) at least 40% of the time between dosing intervals (40% fT > MIC) (Maimongkol et al., 2022), though in neonates with severe infections the higher targets are recommended up to 60% or 100% of time during dosing interval (van Enk et al., 2001; Papp-Wallace et al., 2011; Pacifici, 2019). Due to the recommendations of the French Society of Pharmacology and Therapeutics and the French Society of Anaesthesia and Intensive Care Medicine a free plasma beta-lactam concentration between four and eight times the MIC of the causative bacteria for 100% of the dosing interval (fT ≥ 4–8 x MIC = 100%) is defined as an optimal target for critically ill patients (Guilhaumou et al., 2019). Unbound betalactam’s concentrations exceeding 1- to 4-fold the minimum inhibitory concentration throughout the whole dosing interval (100% fT > 1-4xMIC) are also recommended in other studies for optimal bacterial killing and avoidance of emergence of antimicrobial resistance (Abdul-Aziz et al., 2020).

Meropenem plasma concentrations in preterm newborns are affected by various physiological and pathophysiological factors. Though extracellular distribution of meropenem is predominant, rather high antibiotic concentrations were observed in pulmonary tissues, in pelvic organs, fascia, muscle, omentum, skin (Nicolau, 2008) and brain (Alshaer et al., 2022). Drug distribution varies in adults and neonates; meropenem is a hydrophilic agent, its volume of distribution is dependent on the total water content in the organism. In neonates, especially in preterm infants, there is a higher volume of extracellular fluid and body water content (Ruggiero et al., 2019). Meropenem is mainly eliminated unchanged with urine (up to 70%), and creatinine clearance is usually used as a covariate determining its clearance (Ganguly et al., 2021). For neonates, it is also important to take into account descriptors of maturation of the organism, such as GA, PNA, PCA, postmenstrual age, body weight, etc. (Germovsek et al. (2018); Smith et al., 2011; Ganguly et al., 2021; Touw and van den Anker, 2022).

Variability of PK and PD in preterm newborns emphasizes the need for new PK studies aimed on assessment of population and individual PK parameters of meropenem.

### Aim

The objective of this study was to determine the population pharmacokinetic (PK) parameters of meropenem in preterm newborns from TDM data in real clinical settings and to evaluate the covariates affecting population pharmacokinetic parameters. The estimated meropenem PK parameter values were then used to evaluate PK/PD indices [time above the minimum inhibitory concentration (fT > MIC)] for prescribed meropenem dosage regimens

## Materials and methods

TDM data were collected in real clinical settings (intensive care unit of the City Clinical Hospital 24, Moscow) from population of preterm newborns receiving meropenem therapy to treat bacterial infections. Patients were admitted to the hospital between January 2018 and February 2022. The study was approved by the local Ethics Committee of the Medical Institute of RUDN University (Protocol No. 27 dated 21st of December 2017).

The following criteria were used for exclusion a patient from PK/PD analysis: receiving renal replacement therapy, extracorporeal membrane oxygenation, use of concomitant therapy highly likely interfering with meropenem pharmacokinetics (amoxicillin, ampicillin, azlocillin, cefuroxime, cefotaxime, ceftazidime, oxacillin, ticarcillin, vancomycin, any other carbapenem, furosemide, ibuprofen, valproic acid, probenecid, amphotericin B, indomethacin, ceftriaxone or cefoxitin) within the 3 days prior to TDM procedure. Clinical decisions, patient’s examination, meropenem prescribing, dosage regimen selection and adjustment, drug administration, choice of TDM procedure timing, and taking PK blood probes were carried out by neonatologists and neonatal nurses.

For the PK analysis, demographic and clinical characteristics as well as routinely collected TDM data were retrieved from patients’ records retrospectively.

### PK analysis

Population and individual PK values were estimated by the NPAG program from the Pmetrics package (Leary et al., 2001) based on peak-trough TDM strategy [blood samples taken within 30 min after dosing (peak levels) and just before the next dose (C_trough_)]. A linear one-compartment PK model with zero-order input and first-order elimination was used to fit the meropenem concentration data and to predict PD parameter (fT > MIC of free drug) for different dosage regimens and MIC levels. A one-compartment model was used earlier as a structural model for PK/PD modeling of meropenem in infants (van Enk et al., 2001; Smith et al., 2011; Lima-Rogel et al., 2021; Yonwises et al., 2021). Our model was parameterized as the volume (V) of the central compartment, and the elimination rate constant (Kel). Then using the individualized Bayesian posterior values, patients’ individual values for drug half-life (T1/2) were calculated as 0.693/Kel, total body clearance (CL) was determined as Kel multiplied on V.

For observation weighting for PK modeling, the inverse of the assay variance was used.

The following Schwartz equation for glomerular filtration rate was used:

$$GFR=\frac{k\cdot H}{Scr}$$

Where k = 0.33 for preterm infants, H–height in cm, Scr–serum creatinine in mg/dL, GFR is expressed as mL/min/1.73 m^2^ (Brion et al., 1986).

### Meropenem assay

The concentration of meropenem in human plasma was measured by a high-performance liquid chromatography with ultraviolet detection. The validation procedure followed international practice (Ikeda et al., 2007; Roth et al., 2017). Chromatographic analysis was performed on a Nucleosil C18 column (5 μm, 250 mm × 4.6 mm) and an UV/Vis detector set at 298 nm, by using an isocratic solvent system consisting of water and methanol (85:15, vol/vol), delivered at 1 mL/min. The retention time of meropenem was 6.9 min. The calibration curve was linear between 0.2 and 200 mg/L, with coefficients of determination 0.9996. The lower limit of quantification was 0.2 mg/L. Intraday precision was 1.5%–4.2%, while interday precision was 1.2%–5.3% for each analyte. Sample preparation recovery was at least 85%, regardless of the concentration of meropenem.

### PD analysis

As no exact minimum inhibitory concentration values were available, to calculate fT > 1-4xMIC target values, the EUCAST breakpoints (Susceptible ≤ 2 mg/L, Resistant > 8 mg/L) were used (The European Committee on Antimicrobial Susceptibility Testing, 2021). PK/PD target attainment was defined as unbound concentrations exceeding 2 mg/L or 8 mg/L (8 mg/L or 32 mg/L for 100% fT > 4xMIC) throughout the whole dosing interval. An unbound fraction of 98% was assumed to evaluate the PK/PD target (Drug Bank, 2022).

### Statistical analysis

Statistical analysis was performed with IBM^®^ SPSS^®^ Statistics Version 26.0. Data collection included patient demographic characteristics (gender, age, weight and height at PK sampling), diagnosis, comorbidities, the meropenem dosage regimen(s), concomitant therapy and procedures, serum creatinine at PK sampling, day of meropenem therapy at PK sampling, sampling times and TDM results, adverse events.

Descriptive statistics was presented for demographic and other characteristics, as well as for estimated population PK parameter values. Quantitative data were presented as arithmetic mean ± standard deviation (quartiles Q1, median, Q3) if not stated otherwise. Geometric mean values were used to present statistics for meropenem concentrations, and coefficient of variation (CV%)—for PK parameter statistics.

Categorical variables were summarized as number and percentage of subjects.

Regression analysis was used to evaluate the influence of patient’s covariates (GA, PNA, PCA, body weight, creatinine clearance (GFR) calculated by Schwartz formula, etc.) on estimated individual PK parameters of meropenem.

Spearman rank correlation was used to test the relationships between covariates.

The level of significance was set at an alpha equal to 0.05.

## Results

Demographic, clinical and TDM data of 66 preterm newborns receiving meropenem therapy were included in the analysis (mean ± SD (quartiles Q1, median, Q3): GA was 28.9 ± 2.9 (27.0, 28.5, 31.0) weeks; PNA at blood probe for TDM was 33.0 ± 20.9 (15.0, 30.5, 47.3) days; weight at blood probe was 1.66 ± 0.464 (1.30, 1.58, 2.04) kg; about 61% of patients were male.

Totally, 132 concentrations measured at the first TDM occasion in 66 preterm newborns were included in the meropenem PK population model. For 42(63.6%) patients, TDM was performed within first 4 days of meropenem therapy.

Neonates were hospitalized in ICU with the following diagnoses (one patient could have more than 1): pneumonia (n-56, 84.8%), necrotizing enterocolitis (n-40, 60.6%), congenital infection (n-12, 18.2%), bacterial meningitis (n-6, 9.1%), and bronchopulmonary dysplasia (n −12, 18.2%). Diagnose of COVID-19 was excluded based on medical documentation (negative result of polymerase chain reaction testing).

Demographic and clinical characteristics of patients included in PK/PD analysis are presented in Table 1.

**TABLE 1:** Descriptive statistics for demographic and clinical characteristics.

Data of included patients, n		66
Measured meropenem concentrations, n		132 (66 TDM procedures)
GA, weeks [mean ± SD (quartiles Q1, median, Q3)]		28.9 ± 2.93 (27, 28.5, 31)
PNA at TDM sampling, days [mean ± SD (quartiles Q1, median, Q3)]		33.0 ± 20.88 (15, 30.5, 47.3)
PCA at TDM sampling, weeks [mean ± SD (quartiles Q1, median, Q3)]		33.6 ± 3.69 (30.7, 33.6, 35.0)
Gender, n (%)	Female	26 (39.4)
	Male	40 (60.6)
Weight at TDM sampling, kg [mean ± SD (quartiles Q1, median, Q3)]		1.66 ± 0.464 (1.30, 1.58, 2.04)
Height at TDM sampling, cm [mean ± SD (quartiles Q1, median, Q3)]		38.3 ± 4.26 (35.0, 39.0, 40.3)
Daily dose (mg/day) at TDM sampling [mean ± SD (quartiles Q1, median, Q3)]		94.6 ± 40.4 (54.8, 94.3, 125.8)
Serum creatinine at TDM sampling, µmol/L [mean ± SD (quartiles Q1, median, Q3)]		46.66 ± 20.235 (36.93, 41.15, 54.30)
GFR at TDM sampling, mL/min/1.73 m^2^ [mean ± SD (quartiles Q1, median, Q3)]		27.31 ± 9.676 (19.53, 25.08, 32.53)
Duration of meropenem therapy, days [mean ± SD (quartiles Q1, median, Q3)]		13.9 ± 0.47 (14.0, 14.0, 14.0)
Therapy day at TDM sampling [mean ± SD (quartiles Q1, median, Q3), min, max]		4.2 ± 1.77 (3.0, 4.0, 5.3), min = 2, max = 10

Meropenem was prescribed empirically with daily dose 40–120 mg/kg/day administered by 1–3-h IV infusion 2–3 times a day. Totally, 132 concentrations were included in the meropenem PK population model.

The geometric means of predose and peak concentrations were 3.5 and 23.5 mg/L. In 13 (19.7%) of patients, their C_trough_ levels on the empirical dosage regimens were ≤ 2 mg/L (the S/I EUCAST breakpoint), in 33(50%) infants–below 4 mg/L (intermediate sensitivity), C_trough_ of > 8 mg/L (the I/R EUCAST breakpoint) was reached only in 6(9.1%) patients, C_trough_ of > 32 mg/L was not observed.

**FIGURE 1:**
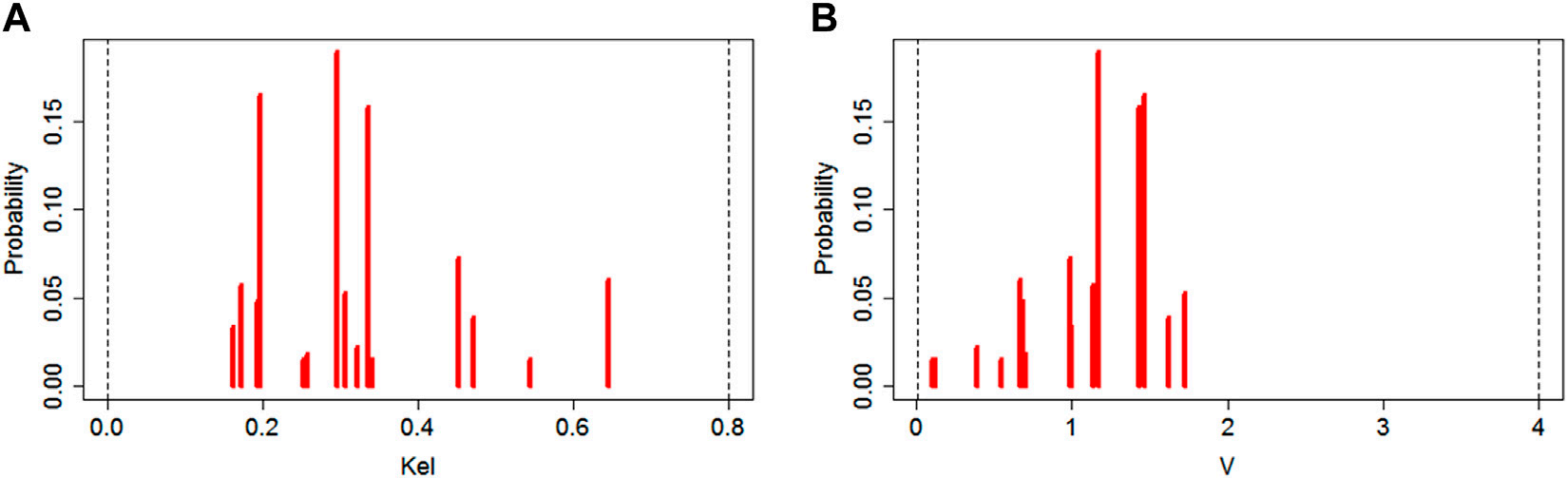
Plot of marginal final cycle parameter value distributions for the linear one-compartment PK model (left side **(A)**—data for Kel; right side **(B)**—data for V).

The population mean ± SD (median) values for constant rate of elimination (Kel) and volume of distribution (V) of meropenem were estimated using NPAG as 0.31 ± 0.13 (0.3) L/h and 1.2 ± 0.4 (1.2) L with interindividual variability (CV, %) of 42% and 33%, respectively. The marginal distributions of these parameter values estimated based on a linear one-compartment model are presented on Figure 1.

The median values for total clearance (CL) and elimination half-life (T1/2) were calculated as 0.22 L/h/kg and 2.33 h with CV = 38.0 and 30.9% (see Table 2).

**TABLE 2:** Descriptive statistics for the calculated PK parameters.

	Mean	Std. Deviation	CV, %	Quartiles		
				Q1	Median	Q3
T1/2, h	2.49	0.769	30.9	1.98	2.33	3.14
V, L/kg	0.75	0.263	35.1	0.56	0.76	0.95
CL, L/h	0.353	0.143	40.5	0.251	0.355	0.469
CL, L/h/kg	0.218	0.083	38.0	0.161	0.219	0.272

**FIGURE 2:**
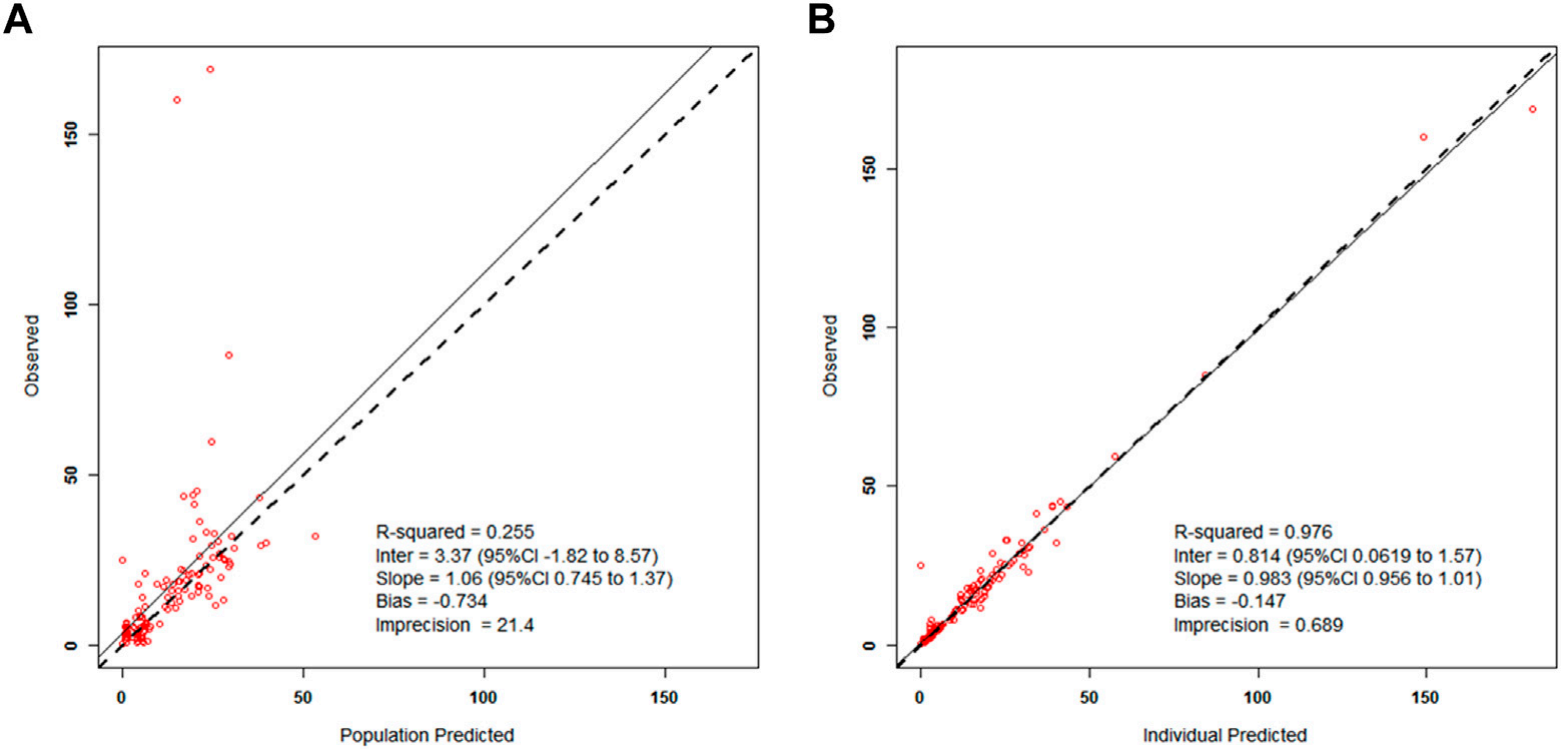
Left side **(A)**—Scatterplot of the predicted–found relationships based on the mean population parameters for all patients; Right side **(B)**—Scatterplot of the predicted–found relationships based on calculating each subject’s own mean value of his/her Bayesian posterior joint parameter density.

Results of the internal validation are demonstrated in Figure 2. Figure 2A demonstrates the wide interindividual variability in patients’ meropenem concentrations when we predicted all patient serum concentrations based on the population mean parameter values. For the same patients, their individual serum levels were predicted based on the mean values of each patient’s Bayesian posterior joint density, as shown in Figure 2B. The quality of prediction was improved, and the regression line in Figure 2B is not significantly different from the line of identity. These model diagnostic results show that the population model by itself gives poor prediction, especially for high concentrations, while the individualized Bayesian posterior models give better prediction (coefficient of determination, R^2^ = 97.6%).

The univariate regression analysis revealed that creatinine clearance, body weight and PCA influenced significantly the meropenem elimination half-life T1/2, and the volume of distribution was mostly correlated with body weight and PCA. But not all observed PK variability can be explained by covariates in the regression models. These statistically significant relationships can explain only from 8 up to 40% of variability (R^2^) in the individual PK parameter values.

**FIGURE 3:**
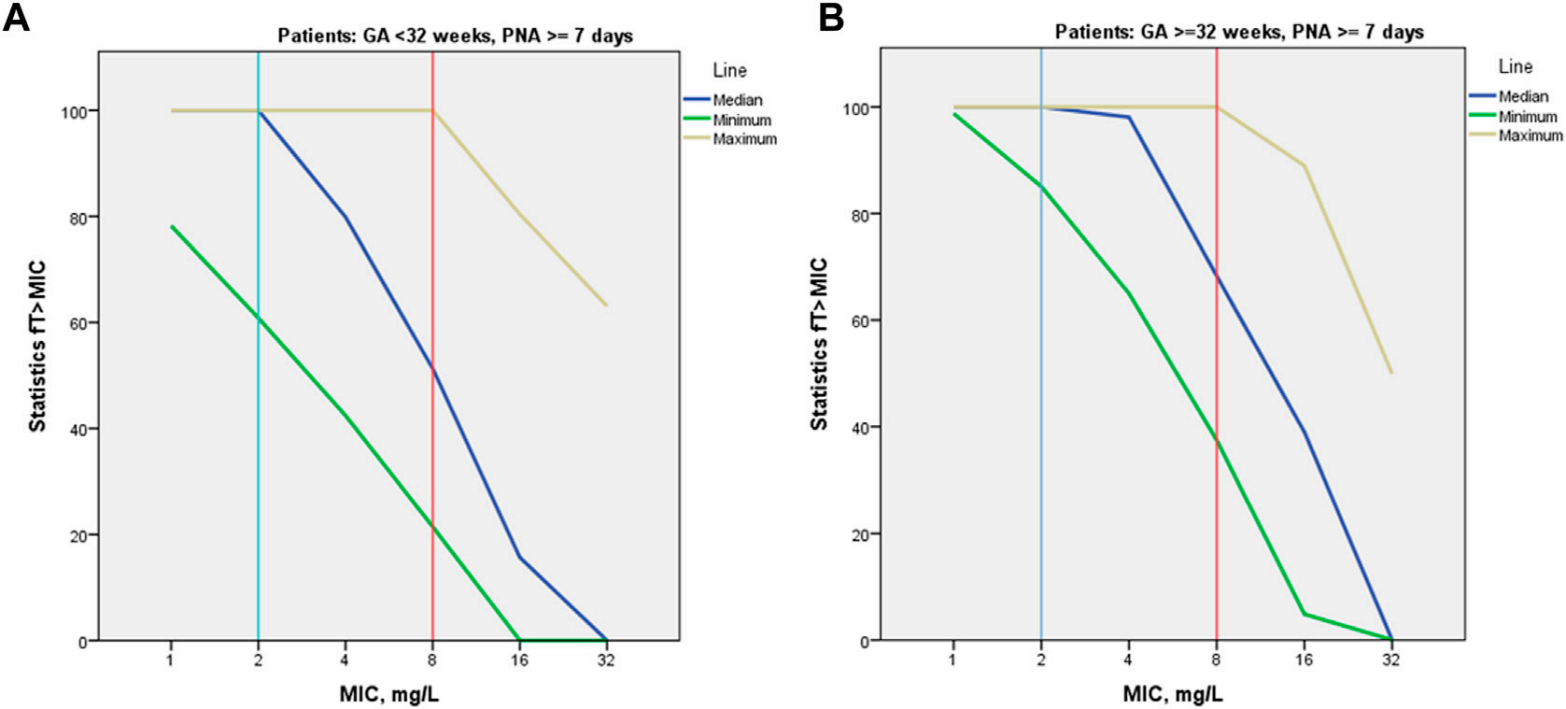
Median values for PK/PD indices 100% fT > 1-4xMIC by MIC and by patient GA group [GA < 32 weeks **(A)** and GA ≥ 32–36 weeks **(B)**].

**FIGURE 4:**
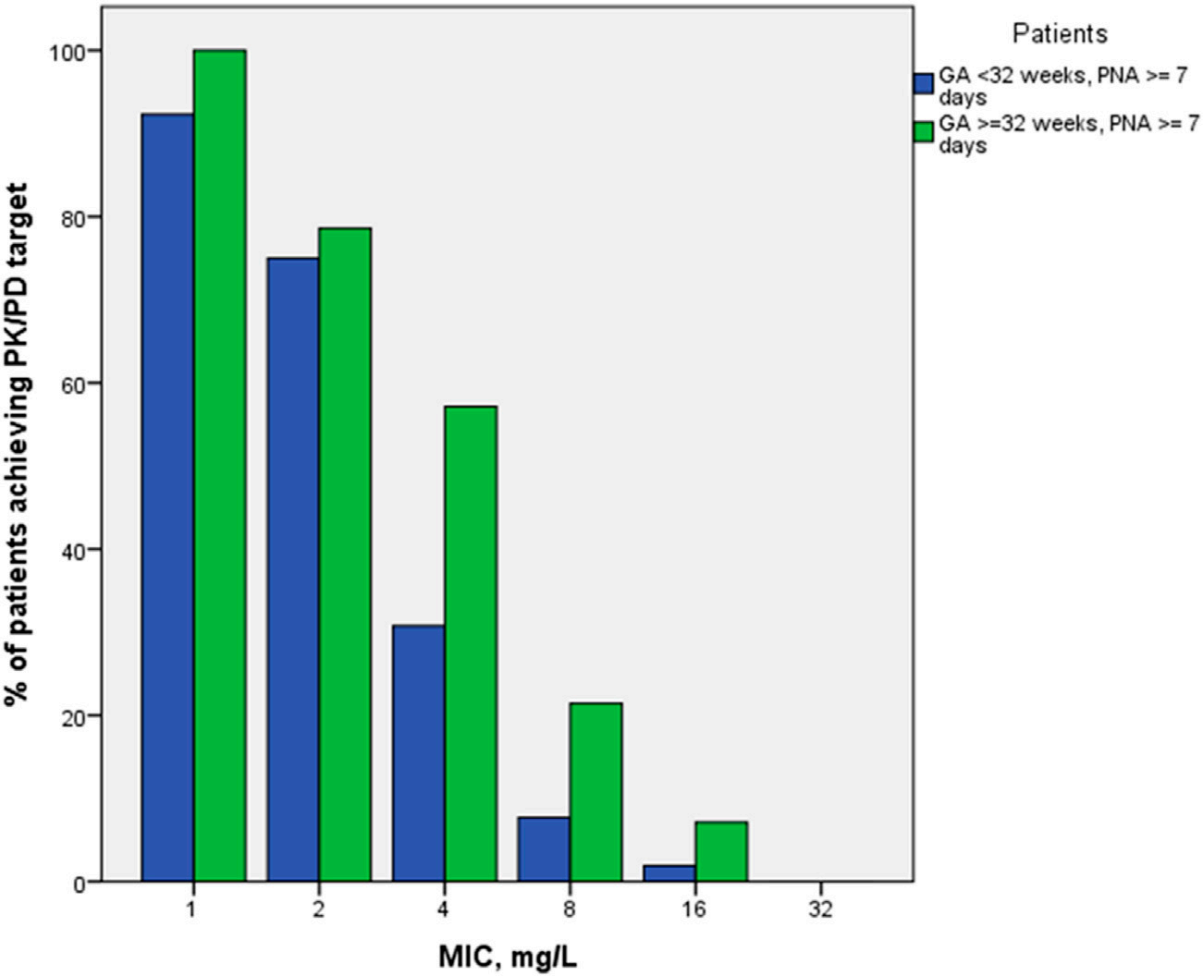
Proportions of patients achieving PK/PD indices 100% fT > 1-4xMIC by MIC and by patient GA group (GA < 32 weeks and GA ≥ 32–36 weeks).

PK/PD indices fT > 1-4xMIC, as well as proportion of patients with 100% fT > 1-4xMIC achievement, were estimated for the empirical meropenem dosage regimens based on estimated individual PK parameter values for the included patients. MIC values of 1, 2, 4, and 8 mg/L were used for calculation of PK/PD target fT > MIC, two extra MIC values of 16 and 32 mg/L were used for evaluation of 100% fT > 4xMIC achievement. For both PK/PD indices, the younger patient group (GA < 32 weeks) had a lower result of achieving the target than the older group (GA ≥ 32–36 weeks) at most MIC values (Figures 3, 4). Achievement of PK/PD target decreased for higher MIC values as organisms become resistant or more aggressive target (4xMIC) applies.

## Discussion

Based on sparse TDM data of 66 preterm newborns and using a population pharmacokinetics approach, meropenem pharmacokinetic characteristics were evaluated. Empirical meropenem dosage regimens were well tolerated by newborns included in the study, no probable drug-attributable clinical or laboratory adverse effects were recorded. High interpatient PK variability was observed.

Our results are comparable with the results previously reported in the literature for neonates. In population of preterm and full-term infants (weight = 2.1 kg). Germovsek E. et al. Found that CL and V were equal to 0.39 L/h and 1.17 L, respectively (Germovsek et al., 2018). In another study (van den Anker et al., 2009), CL value for preterm infants was estimated as 0.2 L/h/kg. For 5 preterm infants, meropenem CL was estimated as 0.16 L/h/kg, V–as 0.61 L/kg (range, 0.38–1.14), and T1/2 was 2.8 h at the fifth day of therapy (van Enk et al., 2001). A lower clearance of 0.12 L/h/kg and V of 0.46 L/kg was reported previously (Smith et al., 2011) for premature and full-term infants. In Bradley et al. (2008) study, the average population CL was 0.104 L/h/kg, and V was 0.4 L/kg (T1/2 = 2.8 h) after a single meropenem dose in preterm and full-term newborns. Similar PK results were reported in the recent study of preterm and full-term newborns (Lima-Rogel V. et al., 2021). A difference in these findings could probably be explained by differences in the study designs, patients’ populations, the PK variability due to disease severity, relatively small sample size of these studies, sparse meropenem sampling in some of them, and PK models used to fit the data.

The values of meropenem PK parameters in our study are different from those reported for adult and older children’s populations. In our study, the median value of serum T1/2 of meropenem was 2.3 h, that is longer than the 1-h estimate in adults (Mouton et al., 2000). In published data T1/2 of meropenem was about 1.6 h in children aged 2–5 months, decreasing to 0.8 h in 6–12 years old children (Blumer et al., 1995), compared to 2.3-h T1/2 found in our study in preterm newborns, indicating renal function maturation. According to previous studies the average meropenem volume of distribution in the young age group was 0.5 L/kg, and in older group–0.4 L/kg (Blumer et al., 1995). In critically ill infants with median age of 7.53 months (interquartile range 1.13–22.50 months), the median estimated weight-normalized meropenem CL and V were 0.23 L/h/kg and 0.4 L/kg, respectively (Yonwises et al., 2021). These age-related changes in PK were probably related to developmental changes associated with renal function and body composition. Our PK results support the previously described tendency (Touw and van den Anker, 2022), that meropenem total body clearance in neonates is comparable with that in adults; however, the volume of distribution in neonates is significantly greater (for weight normalized estimates) (Mouton et al., 2000; Li et al., 2006; Ganguly et al., 2021).

In our study, the most significant descriptors of maturation process were body weight and PCA (highly correlated covariates, Pearson correlation r = 0.84, *p* < 0.001). But each neonate had an individual speed of organ maturation, and pathophysiological changes could potentially increase intraindividual variability. Poor predictable meropenem PK in preterm newborns is a reason for TDM use (Touw and van den Anker, 2022).

Based on PK/PD modeling, recently published studies evaluated recommended meropenem dosage regimens for newborns less than 3 months age and reported fail to meet therapeutic targets in some patients. It was demonstrated that recommended dosage regimens in preterm and full-term infants meet therapeutic targets in at least 83% of subjects (meropenem concentrations > 2 or 4 mg/L for 75% and 50% of the dosage intervals) (Hassan et al., 2020). In another study (Ganguly et al., 2021) for 90% of virtual full-term and preterm newborns (< 3 months of age with complicated intra-abdominal infections) almost all recommended dosing regimens achieved at least a 75% T > MIC for a MIC values ≤ 4 mg/L.

Our data demonstrated that the empirical meropenem dosage regimens (20–40 mg/kg per dose every 8–12 h) do not meet an appropriate attainment for stricter PK/PD targets (100% T > 1-4xMIC) in the included ICU preterm newborns.

The limited studies of PK and PD of meropenem in critically ill neonates suggest that current dosing is frequently inadequate (Touw and van den Anker, 2022). In preterm newborns there are multiple factors affecting PK. Meropenem TDM should be used for optimization of dosage regimen for these patients. For adults, randomized controlled trials demonstrated the benefit of TDM-based dose adjustment of beta-lactam antibiotics for improvement of antibiotic exposure in critically ill and febrile neutropenic patients (De Waele et al., 2014; Sime et al., 2015).

Our results support the opinion (Touw and van den Anker, 2022) that goal-oriented, model-based Bayesian adaptive control based on TDM data can provide quite useful help in optimizing meropenem therapy of newborns.

## Conclusion

Optimal meropenem dosing in premature newborns is challenging due to high interindividual pharmacokinetic variability. A model-based approach can be used in conjunction with TDM data for personalizing meropenem dosage regimen. The estimated population PK model can be used as Bayesian prior information to estimate individual PK parameter values in the preterm newborns and to obtain predictions of desired PK/PD target once the patient’s TDM measured concentration(s) becomes available.

## Limitations

There were limitations in this study. Sparse TDM sampling approach was used in the study and may have limited the ability to investigate multi-compartment PK models. Multivariate covariate analysis was not performed due to the relatively small number of included patients and relatively narrow ranges of values for some covariates. But we believe that these limitations have not precluded the appropriate conclusion.

## Data Availability

The original contributions presented in the study are included in the article/supplementary material, further inquiries can be directed to the corresponding author.
